# Liraglutide Counteracts Endoplasmic Reticulum Stress in Palmitate-Treated Hypothalamic Neurons without Restoring Mitochondrial Homeostasis

**DOI:** 10.3390/ijms24010629

**Published:** 2022-12-30

**Authors:** Haven Griffin, Sarah C. Sullivan, Steven W. Barger, Kevin D. Phelan, Giulia Baldini

**Affiliations:** 1 Department of Biochemistry and Molecular Biology, University of Arkansas for Medical Sciences, Little Rock, AR 72205, USA; 2 Department of Geriatrics, University of Arkansas for Medical Sciences, Little Rock, AR 72205, USA; 3 Department of Neurobiology and Developmental Sciences, University of Arkansas for Medical Sciences, Little Rock, AR 72205, USA

**Keywords:** endoplasmic reticulum, palmitate, obesity, liraglutide, metformin, mitochondria

## Abstract

One feature of high-fat diet-induced neurodegeneration in the hypothalamus is an increased level of palmitate, which is associated with endoplasmic reticulum (ER) stress, loss of CoxIV, mitochondrial fragmentation, and decreased abundance of MC4R. To determine whether antidiabetic drugs protect against ER and/or mitochondrial dysfunction by lipid stress, hypothalamic neurons derived from pre-adult mice and neuronal Neuro2A cells were exposed to elevated palmitate. In the hypothalamic neurons, palmitate exposure increased expression of ER resident proteins, including that of SERCA2, indicating ER stress. Liraglutide reverted such altered ER proteostasis, while metformin only normalized SERCA2 expression. In Neuro2A cells liraglutide, but not metformin, also blunted dilation of the ER induced by palmitate treatment, and enhanced abundance and expression of MC4R at the cell surface. Thus, liraglutide counteracts, more effectively than metformin, altered ER proteostasis, morphology, and folding capacity in neurons exposed to fat. In palmitate-treated hypothalamic neurons, mitochondrial fragmentation took place together with loss of CoxIV and decreased mitochondrial membrane potential (MMP). Metformin, but not liraglutide, reverted mitochondrial fragmentation, and both liraglutide and metformin did not protect against either loss of CoxIV abundance or MMP. Thus, ER recovery from lipid stress can take place in hypothalamic neurons in the absence of recovered mitochondrial homeostasis.

## 1. Introduction

Mice with diet-induced obesity (DIO) and obese humans have neurodegeneration, which is most evident in the hypothalamus [1,2]. In the mediobasal hypothalamus (MBH) of mice with DIO there is pronounced inflammation, gliosis, and neuronal stress mediated by microglia [3,4], as well as endoplasmic reticulum (ER) stress resulting in impaired processing of proopiomelanocortin (POMC) along the secretory pathway of POMC neurons [5,6]. In other regions of the hypothalamus, such as the paraventricular nuclei (PVN), microgliosis is instead undetectable, while there is neuronal loss and decreased expression of ER-synthesized Melanocortin-4 receptor (MC4R) in single-minded family basic helix-loop helix transcription factor 1 (Sim1)/MC4R neurons [7,8]. Importantly, obese humans have increased levels of saturated fatty acids (SFA) such as palmitate in the cerebrospinal fluid [9], and neurotoxic astrocytes have been found to induce neuronal cell death via saturated lipids [10], thereby suggesting that direct exposure to SFA may cause neurodegeneration by targeting the ER. Consistent with that, short-term exposure of rats to a HF diet induces hypothalamic ER stress, which can be reproduced by infusing lipids directly toward the brain (i.e., intracarotidally) [11]. Furthermore, exposure of neuronal cells [12], and immortalized hypothalamic neurons [13] to elevated palmitate has been found to be sufficient to cause ER stress [12]. Importantly, delivery of 4-phenylbutyrate, a chemical chaperone that counteracts ER stress, promotes expression of MC4R in neuronal Neuro2A cells exposed to elevated palmitate [12]. Together, these observations suggest that, in obesity, a target for neuronal injury by increased SFA is ER stress [14]. In ER stress, intracellular signaling pathways, collectively referred to as the unfolded protein response (UPR), are activated to maintain ER function and to promote cell survival [15]. A feature of the UPR is increased synthesis of ER chaperones. However, a recent study finds instead that palmitate treatment does not activate a classic UPR with altered ER proteostasis in human SH-SY5Y neuroblastoma cells [16]. We also found that, in hepatoma cells and liver, palmitate treatment failed to induce a classic UPR with increased level of ER chaperones [17]. These observations suggest that ER stress and adaptive responses are dependent on the type of trigger and on the cell type, highlighting the need to validate outcomes of lipid stress in hypothalamic neurons empirically. In this respect, earlier work has shown that it is possible to derive from the hypothalamus of young mice neurons where specific neuronal subpopulations, such as that of POMC neurons, can be identified by expression of a fluorescently labeled reporter [18]. To find out whether exposure to elevated palmitate induces ER stress, we have derived from the hypothalamus of pre-adult mice primary cultures which maintain hypothalamic neuron cell diversity.

In the hypothalamus of mice exposed to a diet with elevated content of SFA there is, in addition to ER stress, fragmentation of the mitochondrial network in POMC neurons and Sim1 MC4R neurons [7,19,20]. Importantly, experiments using primary cultures show that exposure to elevated palmitate also induces mitochondrial depolarization in dorsal sensory neurons [21], cortical neurons [22], and hypothalamic neurons [7], indicative of direct effects by elevated fat to alter mitochondrial function. Metformin and liraglutide, a glucagon-like peptide 1 receptor (GLP1R) agonist, are the most commonly used drugs to treat obese patients with diabetes mellitus (DM) and pre-diabetes [23,24]. However, the effects of anti-diabetic drugs on modulating ER stress and mitochondrial dynamics in neurons exposed to lipid stress have not yet been studied. Data presented here indicate that liraglutide counteracts ER stress in palmitate-treated hypothalamic neurons better than metformin and does so without restoring mitochondrial homeostasis.

## 2. Results

### 2.1. Liraglutide Prevents Changes in ER Proteostasis and ER Morphology Caused by Palmitate in Neuro2A Cells, while Metformin Is Less Effective

**Figure 1:**
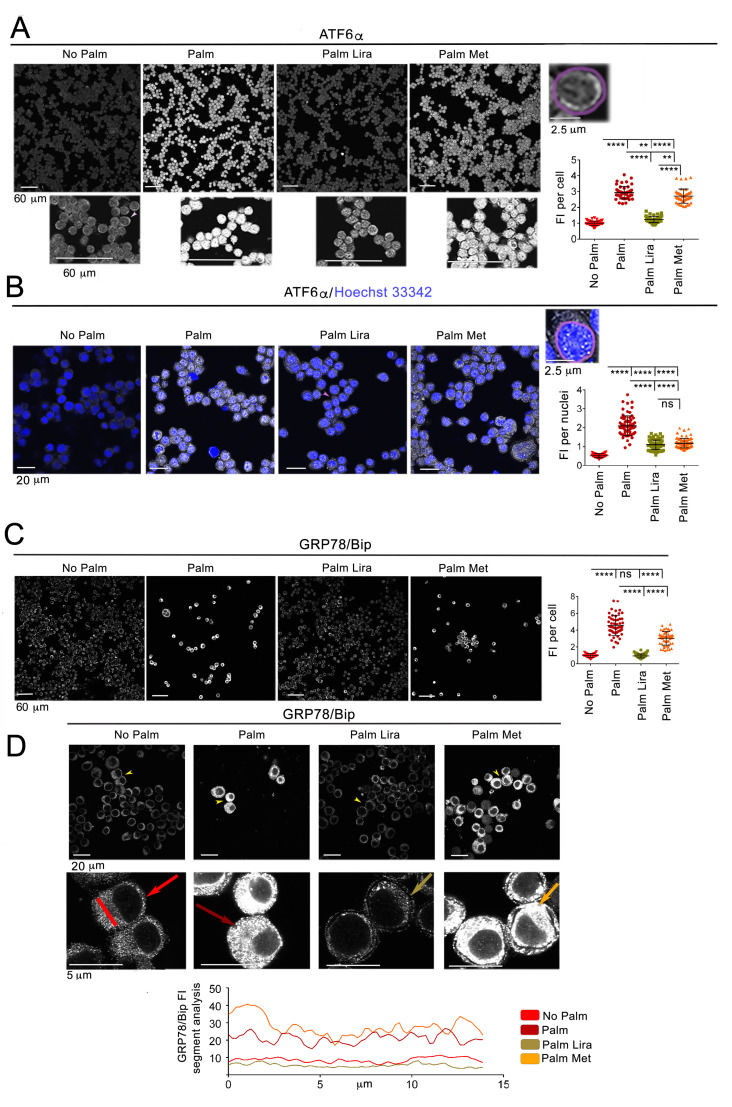
Liraglutide prevents changes in ER proteostasis and ER morphology caused by palmitate in Neuro2A cells. Metformin is less effective in counteracting ER stress. (**A**) Neuro2A cells incubated for 6 h with 250 µM palmitate (Palm) in the presence and absence of 100 nM liraglutide (Lira) and 2 mM metformin (Met) were fixed, immunostained with anti-ATF6α antibody, and imaged by confocal microscopy with the 20× objective of the Olympus FluoviewFV1000 microscope. The graph shows the raw integrated fluorescence intensity (FI) of ATF6α immunostaining within a Region of Interest (ROI) drawn to include the entire cell (magenta line of a representative cell indicated by a magenta arrowhead and shown at higher magnification above the graph, each graph symbol represents one cell, n ≥ 45 cells per condition). (**B**) Neuro2A cells treated as in panel (**A**) were fixed, immunostained with anti-ATF6α antibody, counterstained with the nuclear stain Hoechst 33342, and imaged by confocal microscopy with the 60× objective. Graph shows FI of ATF6α within a ROI drawn to include the nucleus (red line of a representative cell indicated by a red arrowhead and shown at higher magnification above the graph, each graph symbol represents one cell, n ≥ 45 cells per condition). (**C**) Neuro2A cells treated as in panel (**A**) were fixed and immunostained with anti-GRP78/Bip antibody and imaged by confocal microscopy with the 20× objective. Graph shows the FI of GRP78/Bip per cell (n ≥ 25 cells per condition, each graph symbol represents one cell). (**D**) Neuro2A cells treated as in panel (**A**) were imaged with the 60× objective. Yellow arrowheads indicate cells that are shown at higher magnification in the lower panel. Using ImageJ software, a segment was drawn across the cytoplasm of cells treated under the indicated conditions (a representative red segment is drawn in the “No Palm” lower panel. Graphs show the FI values along ImageJ segments drawn in the cells indicated by arrows. ** *p* < 0.01 **** *p* < 0.0001, ns, not significant).

ER stress is counteracted by the UPR, which—rather than constituting stress—represents an adaptive attempt to reduce it [25]. We have found that exposure of neuronal Neuro2A cells to elevated palmitate (250 µM) induces some features of a classic UPR that include splicing of X-box binding protein 1 (XBP1) mRNA to the active transcription factor XBP1s [12]. Another major branch of the UPR is activation of transcription factor 6α (ATF6α), which is cleaved in the Golgi complex to translocate to the nucleus where it functions as a transcription factor to promote synthesis of chaperones [15]. When Neuro2A cells were treated under control conditions and immunostained with an antibody that recognizes both un-spliced and spliced forms of ATF6α [26], the signal was cytoplasmic and barely detectable (Figure 1A). Exposure to elevated palmitate for 6 h increased abundance of ATF6α, which appeared most evident as puncta in the nuclei (Figure 1B). Liraglutide decreased the overall cell abundance of ATF6α, and did so more effectively than metformin. Both liraglutide and metformin blunted to the same extent nuclear accumulation of ATF6α. Thus, exposure to elevated palmitate induces in neuronal cells increased expression and activation of the ATF6α branch of the UPR, and liraglutide and metformin blunt these effects. ATF6α and XBP1 up-regulate the expression of genes encoding ER chaperones such as that of 78-kDa glucose-regulated protein, also referred to as immunoglobulin heavy chain binding protein (GRP78/BiP). When Neuro2A cells were treated with elevated palmitate for 6 h, abundance of GRP78/Bip detected by immunofluorescence microscopy was increased (Figure 1C), thereby indicating altered ER proteostasis. Liraglutide has been found to counteract the increase of GRP78/Bip in Neuro2A cells exposed to ER stress by elevated glucose [27]. Here, we asked whether this is also the case in Neuro2A cells treated with elevated palmitate. Liraglutide prevented the palmitate-evoked elevation of GRP78/BiP, while metformin significantly reduced abundance of this chaperone. Thus, both antidiabetic drugs counteract ER stress in Neuro2A cells exposed to saturated fat, with liraglutide being the most effective. It has been reported that exposure of podocytes to elevated palmitate induces enlargement of the ER [28]. When Neuro2A cells treated under control conditions were observed at higher magnification, GRP78/BiP distribution appeared as small dots of fluorescence over the entire cell cytoplasm (Figure 1D, red arrow). Segment analysis of cells using ImageJ software shows that GRP78/BiP positive dots appeared as barely detectable peaks of fluorescence intensity (FI) over baseline along the segment length (Figure 1D, red line in graph). By contrast, exposure to elevated palmitate changed the distribution of GRP78/BiP immunostaining into larger dots, detected as larger peaks by segment analysis (brown arrow and line in graph). Liraglutide virtually restored ER distribution of GRP78/BiP in palmitate-treated Neuro2A (beige arrow and line in graph), while metformin did not (orange arrow and line in graph). These data indicate that liraglutide, but not metformin, restores ER morphology under conditions of exposure to elevated SFA. 

### 2.2. Liraglutide Increases Abundance and Cell Surface Abundance of Tagged MC4R in Neuro2A_HA-MC4R-GFP_ Cells Exposed to Elevated Palmitate, While Metformin Is Ineffective

**Figure 2:**
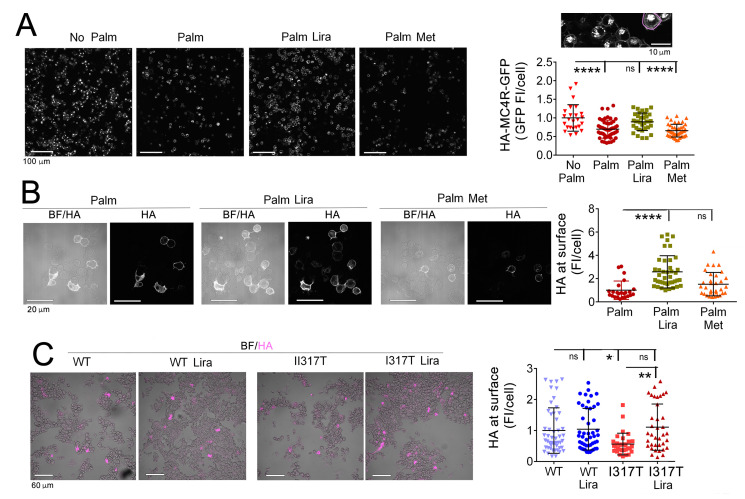
Liraglutide increases MC4R abundance and cell-surface abundance of the receptor in Neuro2A_HA-MC4R-GFP_ cells exposed to elevated palmitate, while metformin is ineffective. (**A**) Live Neuro2A_HA-MC4R-GFP_ cells were incubated for 6 h with 250 µM palmitate (Palm) in the presence and absence of 100 nM liraglutide (Lira) and 2 mM metformin (Met) and imaged by confocal microscopy with the 20× objective. The intrinsic fluorescence intensity (FI) of HA-MC4R-GFP is measured in each cell (encircled by a magenta line in the panel above the graph). (**B**) Neuro2A_HA-MC4R-GFP_ cells were incubated as indicated in (**A**), then fixed as non-permeabilized cells, and immunostained with primary anti-HA antibody. Cells were imaged with the 60× objective. Abundance of HA-MC4R-GFP at the cell surface is monitored by measuring total FI per cell. BF, bright-field microscopy. (**C**) Neuro2A transiently transfected with wt-HA-MC4R-GFP and with wt-HA-MC4R-GFP I317T, respectively, were incubated with no additions, or with the addition of liraglutide. In the graphs, n ≥ 25 cells per condition, each graph symbol represents one cell, * *p* < 0.05; ** *p* < 0.01 **** *p* < 0.0001, ns, not significant.

In the hypothalamus of mice with diet-induced obesity (DIO mice) there is neuronal injury and loss of abundance of melanocortin-4 receptor (MC4R), a GPCR that controls many body functions including energy and glucose homeostasis [29]. Neuro2A_HA-MC4R-GFP_ cells stably express an epitope-tagged receptor carrying HA-tag at the extracellular amino terminus end, and EGFP-tag at the intracellular C-terminus [12]. By using Neuro2A_HA-MC4R-GFP_ cells, we have previously found by Western blot analysis that palmitate decreases abundance of MC4R, thereby modeling in vitro effects by exposure to HF diet observed in neurons in vivo [12]. We reasoned that liraglutide-dependent suppression of ER stress in palmitate-treated Neuro2A cells may also result in improved ER function in protein biosynthesis and increased expression of MC4R at the cell surface. Exposure of Neuro2A_HA-MC4R-GFP_ cells to palmitate for 6 h decreased cell abundance of HA-MC4R-GFP detected by fluorescence microscopy, consistent with earlier results [12]. When liraglutide was applied to Neuro2A_HA-MC4R-GFP_ cells along with palmitate, HA-MC4R-GFP was restored to normal levels (Figure 2A). Conversely, metformin did not reverse the loss of HA-MC4R-GFP induced by exposure to palmitate. In the Neuro2A_HA-MC4R-GFP_ cells exposed to elevated palmitate, liraglutide, but not metformin, also increased cell-surface levels of HA-MC4R-GFP detected by staining with anti-HA antibodies in unpermeabilized cells (Figure 2B). Thus, liraglutide promotes MC4R expression in neuronal cells exposed to elevated palmitate. We found that obesity-linked MC4R variants, such as MC4RI317T, have decreased expression at the cell surface of Neuro2A cells because they are retained as misfolded proteins in the ER and they induce ER stress [30]. When Neuro2A cells were transiently transfected to express wt-HA-MC4R-GFP and mutated HA-MC4R-GFP I317T, liraglutide increased cell-surface expression of the mutated receptor while having no effects on cell-surface abundance of the wt receptor (Figure 2C). These experiments indicate that liraglutide modulates the ER capacity to express wild-type MC4R under conditions of SFA stress and mutated MC4R in the absence of other ER stressors.

### 2.3. Hypothalamic Neurons Derived from Pre-Adult Mice Extend Dendrites with Post-Synaptic Specialization and Include Subpopulations of Sim1 Neurons and Cholinergic Neurons

**Figure 3:**
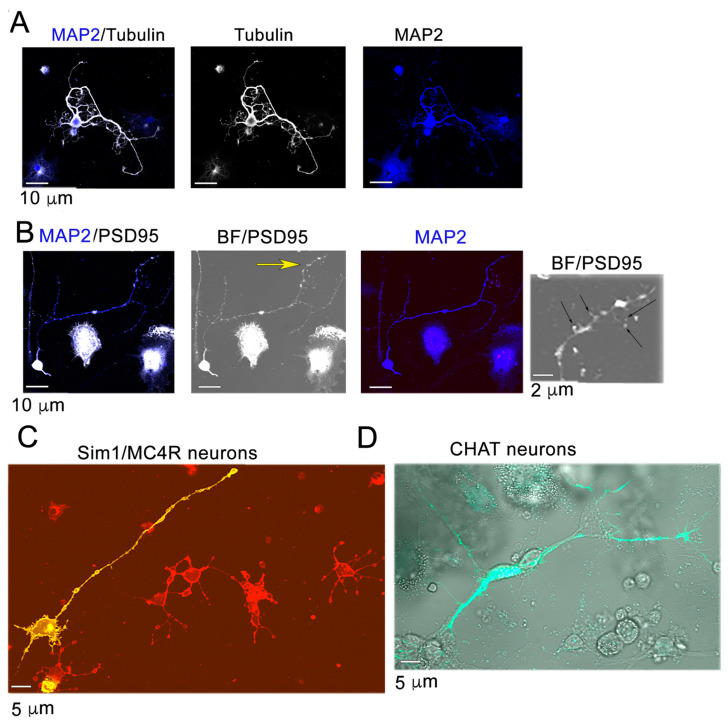
Hypothalamic neurons derived from pre-adult mice extend dendrites with post-synaptic specialization and including subpopulations of Sim1 neurons and cholinergic neurons. (**A**) Primary cultures derived from murine hypothalamus were immunostained with anti-MAP2 (blue fluorescence) and anti-α-tubulin (white fluorescence). (**B**) Cells as in Panel (**A**) were immunostained with anti-MAP2 (blue fluorescence) and anti-PSD95 antibodies (white fluorescence). BF, bright-field microscopy. Yellow arrow indicates region shown at higher magnification. Black arrows indicate PSD95 accumulations at spines. (**C**) The primary culture is derived from Sim1-Cre: Rosa-mEGFP mice to visualize Sim1/MC4R neurons (yellow neurons). (**D**) The primary culture is derived from ChAT-IRES-Cre:CAMPER mice to visualize cholinergic neurons (green neuron).

To study the ER response to SFA in hypothalamic neurons, we used a primary cell culture derived from the hypothalamus of pre-adult mice [7]. Microtubule-associated protein 2 (MAP2) is localized primarily in the cell bodies and dendrites of neurons [31]. All cells in the primary culture expressed MAP2 at the cell body, and there was no detectable signal when immunostaining was carried out with antibodies against glial fibrillary acidic protein (GFAP), an intermediate filament structural protein expressed predominantly in astrocytes [32], and against ionized calcium binding adaptor molecule 1 (Iba1) expressed in microglia [33]. Approximately one fifth of the cells developed processes expressing both MAP2 and the microtubule component α-tubulin [34], indicating that they are dendrites (Figure 3A). In the processes, PSD95 immunostaining was concentrated at the head of small protrusions, indicating that neurons develop dendritic spines with post-synaptic specialization (Figure 3B). The hypothalamus includes numerous neuronal cell types, including Sim1 neurons and cholinergic neurons that control food intake and energy balance [35,36,37]. To determine whether the primary culture mirrors neuronal diversity, we used Sim1-Cre:Rosa-mEGFP mice that express membrane-bound EGFP reporter under the control of the Sim1 promoter [7]. Approximately 5% of the cells that extended processes in the primary culture were Sim1 neurons identified by mEGFP fluorescence (yellow neuron, Figure 3C). Cholinergic neurons of the hypothalamus control food intake [35]. The ChAT-IRES-Cre mice express Cre recombinase under the choline acetyltransferase (*Chat*) promoter [38]. ChAT-IRES-Cre mice were crossed with the CAMPER mouse line [39], where expression of brightly fluorescent TEpacVV is Cre-recombinase-dependent. Approximately 2% of cells in the primary culture from ChAT-IRES-Cre:CAMPER mice expressed TEpacVV and developed processes, indicating that they are differentiated cholinergic neurons. Therefore, the primary culture from pre-adult mice includes multiple neuron subtypes that can differentiate to form dendrites. 

### 2.4. Exposure to Excess Palmitate Disrupts ER Protein Homeostasis in Hypothalamic Neurons and This Effect Is Reversed by Liraglutide, but Not by Metformin

**Figure 4:**
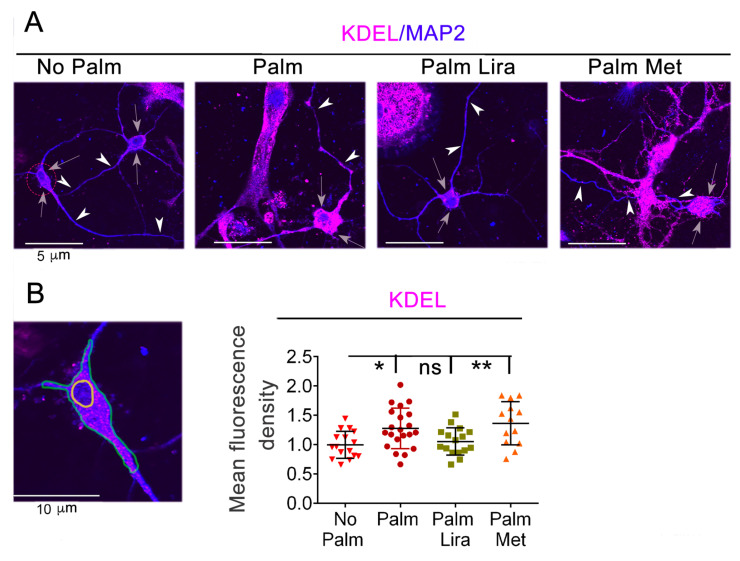
Exposure to palmitate disrupts ER protein homeostasis in hypothalamic neurons. Liraglutide, but not metformin, normalizes ER proteostasis. (**A**) Neurons in primary culture were incubated for 48 h with and without 250 µM palmitate and with and without addition of 100 nM liraglutide (Lira) and 2 mM metformin (Met) to the medium. Cells were fixed, immunostained with anti-MAP2 (blue fluorescence) and anti-KDEL (magenta fluorescence) antibodies, and imaged by confocal fluorescence. Grey arrows indicate cell bodies. White arrowheads indicate neuronal processes. (**B**) Mean fluorescence pixel density of KDEL in a ROI that includes the cytoplasm of the neuron cell body and excludes the nucleus (representative ROI = region within green line that excludes the area encircled by yellow line) by using ImageJ software. The mean pixel density of the ROI is derived from neurons extending processes (n ≥ 13 per condition, each graph symbol represents one cell; * *p* < 0.05; ** *p* < 0.01, ns, not significant).

It has been reported that cultured hypothalamic neurons are resistant to insulin resistance and inflammation induced by 24-h exposure to elevated palmitate, thereby suggesting that neuron injury in obesity involves indirect mechanisms [40]. We extended the time of incubation of the primary hypothalamic neurons with elevated palmitate to 48 h to determine whether effects on ER proteostasis were detectable. ER-resident proteins including GRP78/BiP and other cell chaperones express at the intralumenal site a carboxy-terminal KDEL amino-acid motif, which functions as a signal for retention in the ER [41]. Exposure of hypothalamic neurons to elevated palmitate increased KDEL immunoreactivity (Figure 4), thereby indicating generalized altered ER proteostasis. However, when hypothalamic neurons were exposed to elevated palmitate in the presence of liraglutide, KDEL immunoreactivity remained unchanged as compared to that of the control without excess fat. Conversely, increased KDEL immunoreactivity was still evident when metformin was added to neurons together with the elevated palmitate. These data indicate that exposure of hypothalamic neurons to elevated palmitate alters ER proteostasis, and that liraglutide, rather than metformin, blunts this effect.

### 2.5. Exposure to Palmitate Increases Expression of SERCA2 and This Effect Is Reversed by Liraglutide and Metformin

**Figure 5:**
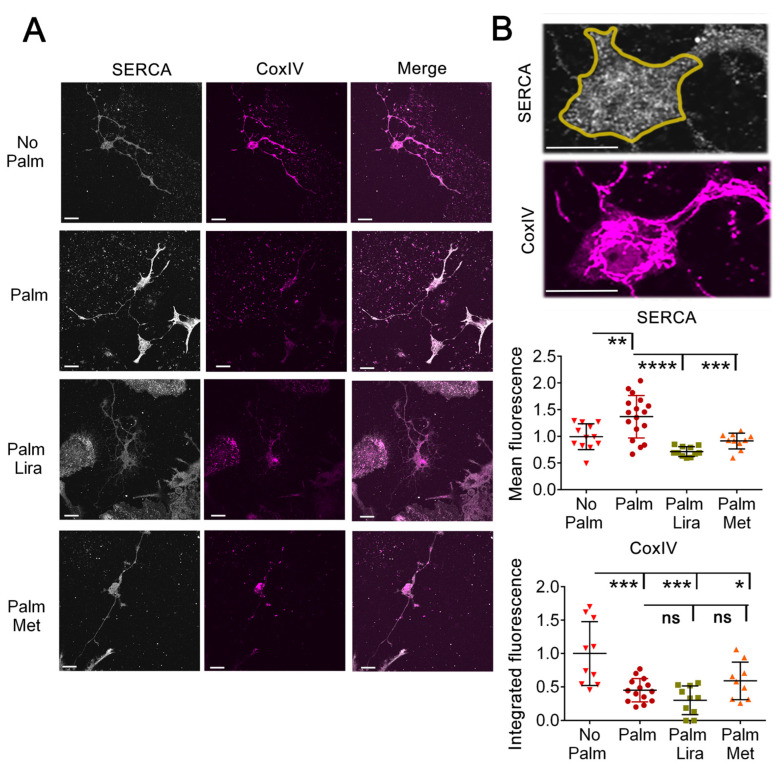
Exposure to palmitate increases expression of SERCA2 and lowers abundance of CoxIV in hypothalamic neurons exposed to elevated palmitate. Metformin and liraglutide normalize abundance of SERCA2, while CoxIV remains decreased. (**A**) Hypothalamic neurons treated as in Figure 4A were immunostained with anti-SERCA2 (white fluorescence) and anti-CoxIV (magenta fluorescence) antibodies, and imaged by confocal fluorescence microscopy. Bars, 5 μm; (**B**) Mean fluorescence pixel density of KDEL and integrated fluorescence density per cell of CoxIV in ROIs that include the cytoplasm (a representative ROI is the area encircled by yellow line in the panel above the graph showing at a higher magnification KDEL and CoxIV immunostaining at the cell body) using ImageJ software. Bars, 5 μm. Pixel fluorescence density of the ROI is derived from neurons extending processes (n ≥ 9 per group, each graph symbol represents one cell; * *p* < 0.05; ** *p* < 0.01; *** *p* < 0.001; **** *p* < 0.0001, ns, not significant).

Dietary fat intake has been found to correlate with a decrease in intraluminal ER Ca^2+^ and the activity of ER Ca^2+^-ATPase 2 (SERCA2) in the liver [42]. Empirical elevation of SERCA levels in the liver alleviates ER stress [43]. On the other hand, it has been reported that ER stress by exposure to tunicamycin increases expression of SERCA2 in PC12 cells [44]. We also observed an increase in SERCA2 levels when hypothalamic neurons were exposed to elevated palmitate (Figure 5). Thus, neurons may respond to SFA-induced reductions in SERCA activity and the accompanying ER stress by elevating SERCA expression. Both liraglutide and metformin reverted SERCA2 to normal levels in neurons exposed to elevated palmitate (Figure 5A,B). Thus, the SFA-associated trigger to induce adaptive increases in SERCA levels appears to have been blunted by both antidiabetic drugs.

Decreased abundance of CoxIV and of other components of the ETC are features of neurodegenerative diseases [45]. We have found that, in the hypothalamus of mice exposed to a high-fat diet, there is loss of cytochrome oxidase IV (CoxIV), the terminal component of the electron transport chain (ETC), and that this effect is reproduced in hypothalamic neurons exposed to elevated palmitate [7]. Here, we asked whether restored SERCA abundance by delivery of liraglutide and by metformin is accompanied by recovered abundance of CoxIV. When added together with the elevated palmitate to the medium of hypothalamic neurons, exposure to either of these drugs did not revert cell loss of CoxIV abundance (Figure 5A,B). Thus, in hypothalamic neurons exposed to elevated palmitate, the ability of liraglutide and metformin to prevent the palmitate-triggered rise in SERCA does not appear to include, or require, maintenance of CoxIV.

### 2.6. Exposure to Palmitate Induces Mitochondrial Fragmentation and Depolarization, Which Are Not Prevented by Liraglutide

**Figure 6:**
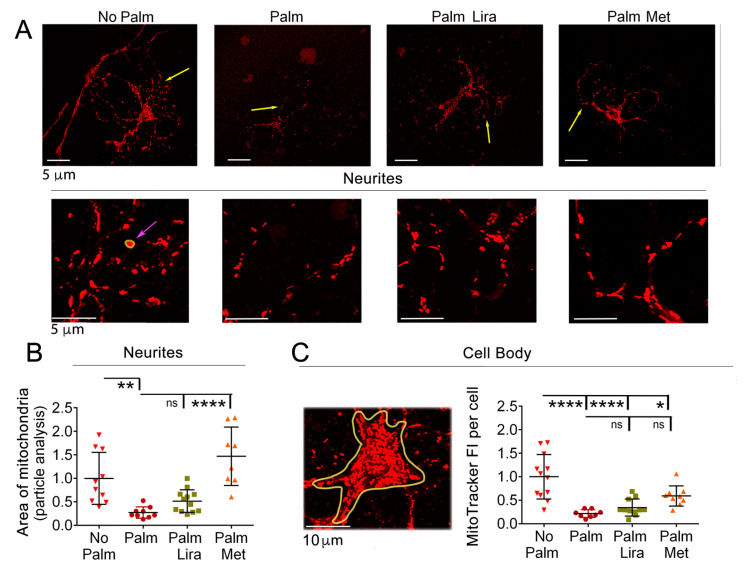
Exposure to palmitate induces mitochondrial fragmentation and depolarization. Metformin and liraglutide do not prevent mitochondrial changes. (**A**) Cells treated as in Figure 4A were incubated for 15 min in the presence of 200 nM Mitotracker Red Red CMXRos, washed, and imaged by live cell microscopy with the 60× objective of the Olympus SV-1000 in complete medium. Neuronal process indicated by yellow arrows are shown enlarged in the lower panels. (**B**) Areas of mitochondria (yellow line encircles one representative mitochondrial area indicated by magenta arrow) were measured along the entire length of at least two processes per neuron by using the “particle analysis” tool of ImageJ. Each symbol in the graph represents the average mitochondrial area per neuron (n ≥ 8 neuron per group, including those shown in panel (**A**), with ~50–100 mitochondria per neuron) measured in ROIs at neurites (a yellow line encircles one representative ROI). (**C**) Integrated fluorescence intensity per cell of MitoTracker in ROIs that include the cytoplasm (a representative ROI is the area encircled by yellow line using ImageJ software). * *p* < 0.05; ** *p* < 0.01; **** *p* < 0.0001, ns, not significant.

In the hypothalamus of mice exposed to a diet with an elevated content of saturated fat there is fragmentation of the mitochondrial network in POMC neurons and Sim1 MC4R neurons [7,19]. Exposure to elevated palmitate for 24 h induces fragmentation of mitochondria, which is detectable at the cell body of insulinoma cells (INS-1 cells) [46]. However, in the hypothalamic neurons in primary culture, mitochondria stained with Mitotracker Red were not individually distinguishable by confocal microscopy at the cell body, making it difficult to measure their individual sizes by using the “particle analysis” tool of ImageJ. However, in the neuronal processes, mitochondria appeared as distinct particles and were therefore amenable to measurement (Figure 6A). Addition of elevated palmitate to the neuronal culture decreased the average size (area) of mitochondria (Figure 6B). Delivery of metformin to diabetic mice has been found to inhibit mitochondrial fragmentation and to counteract injury in endothelial cells [47]. Consistent with that, the addition of metformin to hypothalamic neurons exposed to elevated palmitate prevented any decrease in average mitochondrial size. Conversely, liraglutide failed to impact the effects of palmitate on mitochondrial size. 

Mitotracker Red is a weak base that accumulates in mitochondria depending on proton gradient across the inner mitochondrial membrane [48]. When hypothalamic neurons were treated with palmitate, Mitotracker Red fluorescence intensity (FI) at the cell body was decreased, indicating loss of MMP (Figure 6A,C), consistent with our previously published results [7]. Mitotracker Red FI was not restored when either liraglutide or metformin were added to the medium together with palmitate. Thus, exposure of hypothalamic neurons to SFA induces mitochondrial depolarization, and this effect persisted in the presence of either liraglutide or metformin.

## 3. Discussion

The main findings of this paper are as follows: (1) exposure to elevated palmitate is sufficient to alter ER protein abundance in primary hypothalamic neurons; (2) the antidiabetic drug liraglutide is more effective than metformin in normalizing ER proteostasis, morphology, and protein synthesis in hypothalamic neurons and neuronal cells exposed to SFA; (3) the effects of liraglutide to blunt ER stress in neurons exposed to SFA do not require restored mitochondrial dynamics and MMP. 

It has been recently found that a commonly used antidiabetic drug, metformin, attenuates ER stress markers in pancreatic beta-cells exposed to palmitate [49] and that metformin and liraglutide have similar effects in decreasing markers of the UPR that include abundance of GRP78/BiP in coronary artery endothelial cells exposed to stress by elevated glucose [50]. However, treatment with metformin did not blunt the increase in abundance of GRP78/BiP in peripheral blood mononuclear cells of patients with type-2 DM [51], thereby indicating unresolved ER stress. Here, by using transformed neuronal cells in culture, we compared the ability of metformin and liraglutide to counteract ER stress by SFA exposure. We found that liraglutide, more effectively than metformin, blunts SFA-induced increase in abundance of ATF6α and of GRP78/BiP in Neuro2A cells, suggesting recovered ER function. An outcome of resolved ER stress is the recovered ability to synthesize proteins such as MC4R, the abundance of which is decreased in the hypothalamus of obese mice [7]. Here, we find that liraglutide, rather than metformin, increases abundance and cell-surface localization of MC4R, indicating that the resolved ER proteostasis by liraglutide is accompanied by recovered ER function. Another feature of ER stress is dilation of ER cisternae [52], such as that taking place in hepatoma cells expressing mutated alpha [1]-antitrypsin Z (ATZ) [53,54]. Data presented here indicate that exposure of Neuro2A cells to elevated palmitate induces morphological changes in the ER, with formation of enlargements containing increased abundance of ER chaperones. On the other hand, stress resolution is marked by a return of the ER to normal size [55]. Liraglutide, but not metformin, protects ER morphology under conditions of exposure to elevated SFA. It is concluded from these data that liraglutide has more potent protective effects toward the ER under lipid stress in neuronal cells. 

In vitro studies by using neuronal cells and immortalized hypothalamic lines to determine whether exposure to elevated SFA alters ER proteostasis have given contradictory outcomes [12,13,16]. We have used here, for the first time, physiologically relevant primary cultures of hypothalamic neurons to validate outcomes by exposure to elevated SFA observed in neuronal cells. Earlier work found that hypothalamic neurons derived from young mice include the subpopulation of POMC neurons, which differentiate in culture to form functional autapses [18]. Similarly, we find here that hypothalamic neurons derived from pre-adult mice form post-synaptic specialization at dendritic spines and reflect the neuronal diversity of the hypothalamus by including Sim1 neurons [56] and cholinergic neurons [38]. We used the entire population of hypothalamic neurons able to extend processes for the analysis. Using this system, we concluded that exposure to elevated SFA alters ER proteostasis in hypothalamic neurons. Peripherally delivered GLP1R agonists appear to have direct access to multiple regions of the brain including the hypothalamus [57]. Consistent with that, it has been reported that GLP1R expressed in neurons mediates liraglutide’s ability to decrease body weight in mice with DIO [58] and that GLP1 directly stimulates POMC neurons in the arcuate nucleus to decrease body weight [59]. Our data indicate that the majority of hypothalamic neurons in primary culture exposed to elevated SFA respond to liraglutide, rather than to metformin, by re-establishing ER protein homeostasis. Thus, liraglutide may be more effective than metformin in counteracting ER stress in hypothalamic neurons exposed to lipid stress. In conclusion, outcomes from this work suggests liraglutide, rather than metformin, as the most effective drug to counteract ER stress resulting from SFA stress.

Physiologically, SERCA functions to maintain low levels of cytosolic Ca^(2+)^, and elevated level of intraluminal Ca^(2+)^ [60]. ER stress in neurodegeneration by amyotrophic lateral sclerosis has been found to affect Ca^(2+)^ handling and protein folding in neurons [61]. We find here that hypothalamic neurons exposed to SFA have increased abundance of SERCA2, consistent with an earlier finding that PC12 cells with drug-induced ER stress also have increased SERCA abundance and calcium-pumping capacity [44]. It has been found that overexpression of SERCA in the liver of mice with DIO reduces chronic ER stress [62]. Exposure to elevated SFA has also been found to increase PC/PE ratio in liver cells [62] and neuronal cells exposed to elevated palmitate [16]. These data suggest the possibility that, in neurons, increased abundance of SERCA is part of an adaptive ER response to counteract stress induced by elevated palmitate. We find that liraglutide and metformin treatment prevent disturbances in the abundance of SERCA, suggesting that the drugs may blunt triggers of ER stress, such as altered ER membrane phospholipid composition. However, in hypothalamic neurons exposed to palmitate, metformin’s ability to prevent SERCA elevation was not paralleled by normalization of ER proteostasis. Thus, in neurons exposed to lipid stress, changes underlying normalized SERCA expression by both liraglutide and metformin may be necessary, but are not sufficient, to correct ER dysfunction.

Dysregulation of astrocyte and microglia function, including that induced by ER stress, contributes to neurological decline in CNS diseases [63,64]. Neurodegeneration in the hypothalamus of obese mice exposed to HF diet is accompanied by microgliosis, which is most evident in the arcuate nucleus of males [8,65] and is virtually undetectable in the PVN of both male and female mice [8]. It has been recently proposed that classically activated neuroinflammatory microglia induces activation of astrocytes, which in turn release SFA to induce neuronal cell death [10]. Thus, it is possible that, under conditions of HF diet, neurodegenerative events result from excess SFA produced by astrocytes or derived directly from the circulation because of changes in permeability of the brain-blood barrier [66]. Results presented in this paper indicate that direct exposure to SFA causes ER stress in hypothalamic neurons and that liraglutide counteracts this effect more effectively than metformin. A future direction of this research will be to compare the abilities of liraglutide and metformin to counteract ER stress in neurons and astrocytes in the mediobasal hypothalamus, PVN, and other hypothalamic regions of male and female mice treated with HF diet. In conclusion, the indication provided in this work that liraglutide is more potent than metformin to reduce ER stress in neurons may extend to astrocytes and may have implications for pharmacological prevention of hypothalamic neurodegeneration in obesity.

It has been found that in mice exposed to high-fat diet there is ER stress and mitochondrial dysfunction with loss of mitochondria–ER contact sites in POMC neurons [19,20]. In Sim1/MC4R neurons of the PVN, loss of MC4R is concomitant with fragmentation of mitochondrial network [7]. In vitro experiments have found that direct exposure to elevated palmitate induces fragmentation of mitochondria in insulinoma cells [46], podocytes [28], epithelial cells [67], and fibroblasts [68]. Similarly, we find here that direct exposure to elevated palmitate is sufficient to induce mitochondrial fragmentation and depolarization in hypothalamic neurons. Exposure to increased glucose load also results in mitochondrial fission and reduced generation of reactive oxygen species in ventromedial hypothalamic neurons, and this effect is mediated by dynamin-related peptide 1 (Drp1) under the control of uncoupling protein 2 (UCP2) [69]. Interestingly, metformin inhibits Drp1 activity in peripheral tissues [47]. On the other hand, inhibition of Drp1 blunts mitochondrial fission and fatty-acid oxidation in fibroblasts under conditions of palmitate exposure [67]. Consistent with the ability of metformin to inhibit mitochondrial fission, we find here that the drug protected against shrinkage of average mitochondrial area in neurons exposed to palmitate, while liraglutide does not. Conversely, under these conditions liraglutide maintains ER protein homeostasis while metformin does not. Thus, our findings suggest that liraglutide’s inability to prevent mitochondrial fragmentation and depolarization is inconsequential; it may instead promote ER recovery in hypothalamic neurons under lipid stress. For instance, it has been proposed that uncoupled respiration in pancreatic beta-cells is a mechanism for removing excess nutrients [70] and that mild impairment of mitochondrial oxidative phosphorylation is beneficial in treating obesity [71]. 

The finding that liraglutide inhibits aspects of the UPR, re-establishes proteostasis, and promotes expression of MC4R in neuronal cells opens up the question of the mechanism by which the drug acts. The most extensively characterized signal transduction pathway connected to GLP1R is a G-protein containing Gα_s_, which stimulates adenylate cyclase and the cyclic AMP cascade, including protein kinase A (PKA), however, the receptor may also activate PI3 kinase, ERK, and protein kinase C signaling pathways [72]. Nevertheless, PKA has been implicated in the ability of incretins to decrease expression of cluster of differentiation 36 (CD36) [73]. CD36 is expressed in hypothalamic neurons, where it acts as a major regulator of neuronal fatty acid sensing [74]. CD36 has been implicated in palmitic acid-induced lipotoxicity in Neuro2A cells [75]. Thus, a possible mechanism by which liraglutide blunts ER stress is by decreasing expression of CD36 or other proteins that can facilitate the transport of SFA into cells. ER stress is associated with increased protein misfolding, which can initiate the generation of reactive oxygen species (ROS) cascades involved in neurodegenerative diseases [76]. GLP1R agonists such as liraglutide have been found to reduce the level of free radicals by promoting expression of anti-oxidative factors such as superoxide dismutase (SOD) and catalase [72]. Consistent with an anti-oxidative role of liraglutide in neurons, another GLP1 agonist, Exentin-4, has been found to inhibit the generation of ROS in a neuronal cell line exposed to elevated palmitate [22]. Thus, a possible neuroprotective mechanism of action of liraglutide is the induction of anti-oxidative responses to blunt vicious cycles whereby protein misfolding in the ER increases generation of ROS, further impairing ER proteostasis, calcium handling, and mitochondrial function [77].

In conclusion, while it is known that neuronal injury takes place in the hypothalamus in the context of diet-induced obesity [7,8,78,79], the mechanisms of such injury and effects of current antidiabetic drugs to counteract neurodegeneration are mostly unknown. The work presented here indicates that in hypothalamic neurons elevated SFA can cause altered ER proteostasis, which is blunted more effectively by liraglutide than by metformin without requiring normalization of mitochondrial parameters.

## 4. Materials and Methods

### 4.1. Chemicals

Formaldehyde (Cat # BP531-500), Corning Penicillin/Streptomycin 50× (Cat # 30-001-CI), Corning^®^ 100–1000 µL Universal Fit Racked Pipet Tips (Cat # 07-200-304), AccuTec Blades™ Personna Single-edge Prep Razor Blades (Cat # 12-640-18), Corning Falcon^®^ 70 µm Cell Strainer (Cat # 08-771-19), and 18 × 18 mm Fisherbrand™ Premium Cover Glasses (Cat # 12-548-AP), Fisherbrand™ Premium Frosted Microscope Slides (Cat # 125442) were from ThermoFisher Scientific (Waltham, MA, USA). Neurobasal™-A Medium (Cat # 10888022), B-27™ Plus Neuronal Culture System (B27 Plus Neuro) (Cat # A3653401), MitoTracker™ Red CMXRos (MitoTracker Red, Cat # M7512), Lipofectamine™ 3000 Transfection Reagent (Cat # L3000015), Hoechst 33342 (Cat # H1399) and Hank’s Balanced Salt Solution (HBSS, Cat # 14170112) were purchased from ThermoFisher Scientific (Waltham, MA, USA). Fetal Bovine Serum (FBS) (Cat # FP-0500-A) was purchased from Atlas Biologicals (Fort Collins, CO, USA). Papain (Cat # LS0031191) and Earle’s Balanced Salt Solution (EBSS, Cat # LK003188) were purchased from Worthington Biochemical Corporation (Lakewood, NJ, USA). Glass-Bottomed 35mm Cell Culture Dish (Cat # 801002) were from Southern Labware (Cumming, GA, USA). Recombinant mouse FGF2 protein (Cat # ab229521) and recombinant human FGF1 protein (Cat # PHR1084 ab222361) were purchased from Abcam (Cambridge, MA, USA). Metformin hydrochloride (Cat # PHR1084 and 1α,25-dihydroxyvitamin D3 (vitamin D3, Cat # D1530), albumin from chicken egg white (Cat # A5503), essentially fatty-acid-free bovine serum albumin (Cat # A7511), sodium palmitate (Cat # P9767), DNase I (Cat # 11284932001), Cat # Poly-L-lysine solution (Cat # P4707), Triton X-100 (Cat # X100-100ML) were from Millipore Sigma (Burlington, MA, USA). Melanotan II (MTII, Cat # 2566) and liraglutide (Cat # 6517) were from Tocris (Minneapolis, MN, USA). Primary antibodies: chicken anti-MAP2 Antibody (Cat # NB300-213) and ATF6 (Cat # 70B1413.1) were from Novus Biologicals (Minneapolis, MN, USA); rabbit anti-PSD95 (Cat # ab18258), rabbit anti-COX IV antibody (Cat # ab16056), rabbit anti-GRP78/BiP (Cat # ab21685), rabbit anti-α-tubulin (Cat # ab18251) and goat anti-GFAP antibody (Cat # ab53554), from Abcam (Cambridge, MA, USA); mouse anti-KDEL antibody (Cat # ADI-SPA-827) from Enzo (Farmingdale, NY, USA); rabbit C29F4 anti-HA (Cat # 3724) antibody from Cell Signaling Technology (Danvers, MA, USA); and mouse SERCA2 Antibody (Cat # sc-376235) from Santa Cruz Biotechnology (Dallas, TX, USA). Rabbit polyclonal anti-Ionized calcium-binding adaptor molecule 1 (Iba1) antibodies (Cat# 019-19741) was from Wako Pure Chemical Industries, Ltd (Richmond, VA, USA). Secondary antibodies: Cy™5 AffiniPure Donkey Anti-Rabbit IgG (Cat # 711-175-152); DyLight™ 405 AffiniPure Donkey Anti-Rabbit IgG (H+L) (Cat # 711-475-152); DyLight™ 405 AffiniPure Donkey Anti-Chicken IgY (IgG) (H+L) (Cat # 703-475-155); Alexa Fluor^®^ 647 AffiniPure Donkey Anti-Mouse IgG (H+L) (Cat # 715-605-151) were from Jackson Immuno Research Inc. (West Grove, PA, USA). Neuro2A cells (Cat # CCL-131) were from ATCC (Manassas, VA, USA).

### 4.2. Neuro2A Cell Culture and Treatment with Palmitate, Metformin, and Liraglutide

Neuro2A cells and Neuro2A cells stably transfected with HA-MC4R-GFP (Neuro2A_HA-MC4R-GFP_ cells) [80] were cultured in DMEM with 10% fetal bovine serum (FBS) and 5% penicillin/streptomycin (growth medium) as previously described in 35-m plates containing 18 × 18 mm glass coverlips pre-treated with poly-lisine solution for 2 h [80]. To treat cells with palmitate, 5 mM palmitate in 5% FA-free BSA was prepared as previously described [12]. Briefly, palmitate stock was prepared as 100 mM sodium palmitate in 0.1 M NaOH heated to 72 °C. Essentially FA-free BSA was prepared in water as a 10% sterile solution. To obtain the 5 mM palmitate/BSA stock, 500 μL of 100 mM palmitate stock (72 °C) was pipetted into 9.5 mL of BSA kept at 37 °C. The palmitate/BSA solution was aliquoted and stored at −20 °C. To defrost, aliquots were placed in a dry bath incubator set at 56 °C. Metformin was prepared as a 100 mM (50×) sterile stock solution in water at kept at 4 °C. Liraglutide was prepared as a 25 mM (250×) sterile stock solution in water and kept as frozen aliquots. To treat cells, liraglutide, metformin and palmitate were directly added to the complete medium.

### 4.3. Cell Transfection

Plasmids expressing wt-HA-MC4R-GFP and wt-HA-MC4R-GFP I317T in the vector pEGFP-N2 were prepared as previously described [30]. Transfection was carried out using subconfluent dishes (35-mm diameter) of Neuro2A cells plated 18–24 h before the procedure. Transfection was carried out using 0.6 μg of plasmid, 3 μL of Lipofectamine 3000 and 4.5 μL of P3000, and following the vendor’s procedures. Approximately 18 h after transfection, cells were trypsinized and plated into two 35-mm diameter dishes with poly-lysine treated coverslips. Approximately 24 h after plating, cells were treated with liraglutide, metformin and palmitate, as indicated in the figure legends.

### 4.4. Immunofluorescence and Fluorescence Microscopy of Neuro2A Cells

The medium of Neuro2A cells incubated in different conditions was pipetted out of the plate and cells were fixed with a solution of freshly prepared 4% formaldehyde in PBS for 30 min at room temperature.

#### 4.4.1. Immunofluorescence Microscopy of Permeabilized Neuro2A Cells

After fixation, cells were washed with PBS thrice, and incubated with PBST containing Sodium Azide 0.01% (*w/v*), chicken egg albumin (50 μg/mL), and 0.2% Triton X-100 for 30 min (PIF buffer). Cells were then incubated in PIF containing primary antibodies for 2 h at room temperature, washed three times with PIF and incubated in PIF containing fluorescent labeled secondary antibodies for 2 h at room temperature, washed three times with PIF, and once with PBS, before adding mounting medium and setting the coverslip onto microscope slides. Fluorescence microscopy was carried out using the confocal Olympus FV1000 microscope (Center Valley, PA, USA). To measure abundance of GRP78/Bip, cells were imaged with the 20× objective. Using the ImageJ software, the raw integrated fluorescence intensity of immunostaining in each cell was measured within a ROI drawn to include individual cells. To measure changes in the morphology of the ER cells were instead imaged with the 60× objective and distribution of the immunostaining was monitored using the “segment” tool of ImageJ.

To measure cell and nuclear abundance of ATF6, Neuro2A cells were plated onto polylysine-treated glass-bottomed culture dishes, treated with palmitate, metformin, and liraglutide as described above, and fixed in methanol at 4 °C for 15 min. Immunostaining was carried out as described above, but using for all steps detergent-free IF buffer (PBS containing Sodium Azide 0.01% (*w/v*) and chicken egg albumin (50 μg/mL)). Nuclei were stained using Hoechst 33342 and following the manufacturer instructions.

#### 4.4.2. Immunofluorescence Microscopy of Un-Permeabilized Neuro2A Cells

Cells were washed with PBS thrice, and incubated with IF buffer. Immunostaining was carried out as above, with the exception that all steps were carried out using PBF/IF buffer.

#### 4.4.3. Fluorescence Microscopy of Neuro2A_HA-MC4R-GFP_ Cells

Neuro2A_HA-MC4R-GFP_ cells were plated in 35 mm cell-culture dish with glass bottom and treated as described in “Figure Legends”. Cells were transferred onto the stage of confocal Olympus FV1000 microscope and imaged using the 20× objective. ImageJ software (ImageJ 2.1.0; Java1.8.0_172) was used to measure the raw integrated fluorescence intensity of EGFP in each cell.

### 4.5. Animal Strain, Animal Care, and Diet

The C57BL/6J mice were obtained from Jackson Laboratories (Cat # JAX:000664). All mice were bred and housed in the University of Arkansas for Medical Sciences (UAMS) vivarium. Mice were housed in a temperature-controlled environment with 12-h:12-h light-dark cycle, “lights on” at 06:00 h and “lights off” at 18:00 h and given ad libitum access to food and water. Each cage housed 2–5 mice and had the addition of one “mouse hut” per cage for enrichment. UAMS veterinarians checked on the mice daily to monitor their health. Mice were weaned at 21 days of age. Sim1-Cre:Rosa-mEGFP mice with expression of membrane-bound EGFP (mEGFP) in Sim1 neurons were obtained as described [8]. The ChAT-IRES-Cre mice expressing Cre recombinase under the choline acetyltransferase (Chat) promoter [38] were obtained from Jackson Laboratories (Cat # JAX:006410) and crossed with CAMPER mice from Jackson Laboratories (Cat # JAX:032205) [39] to obtain ChAT-IRES-Cre:CAMPER. The DNA used for genotyping was extracted from a small piece of tail (<2 mm). Male and female mice (4–6-weeks old) were euthanized by CO_2_ asphyxiation to harvest the hypothalamus.

### 4.6. Preparation of Primary Hypothalamic Neurons

All solutions, sterilized by passage through 0.22-μ filters, were equilibrated with 95% O_2_, 5% CO_2_. All glasswares, plasticwares and instruments were sterile. Brains were extracted and individually placed in 24-well plate with 0.5 mL of ice-cold solution of Neurobasal A Medium containing Penicillin/Streptomycin 100 U/mL (Neurob-A P/S medium). For each brain, a section of approximately 2 mm thickness was cut using a Stoelting stainless steel brain matrix, with one single-edge blade placed anterior to the optic chiasm and the other blade placed anterior to the pons. From the coronal slice, a tissue rectangle of 2 mm width and 3 mm height centered around the entire 3rd ventricle to include the mediobasal hypothalamus and the PVN was dissected. The section was further cut by using single-edge blades into cubes of approximately 0.2 mm sides. The minced tissue was placed in an individual tissue culture well containing 0.5 mL EBSS with papain 20 U/mL, 1 mM EDTA, and 1 mM DTT. The mixture was incubated in a tissue culture cabinet equilibrated with 95% air and 5% CO_2_ for 15 min at 37 °C under agitation (OrbiShaker™ MP Orbital Microplate Shaker, 500 rpm), then DNAase was added from 1% *w/v* stock solution in EBSS to reach a final concentration of 0.005% *w/v*, and the mixture was further incubated in the tissue culture cabinet for another 15 min at 37 °C under continuous agitation. After digestion, the minced brain tissue in each well was passed once through a 1 mL pipet tip, and pipetted into Cell Strainer with 70 μm pores and placed onto a 6-well plate. FBS was added to cells to reach a final concentration of 10% (*v/v*). The filtered cells were pipetted onto glass-bottomed culture dishes and treated for 2 h with poly-lysine. The plates were transferred to the cell-culture cabinet without agitation to allow for cells to adhere to the glass bottom for 30 min. Then 0.5 mL of Neurob-A P/S medium containing FBS was added and cells were further incubated in the tissue culture cabinet for 1.5 h. The unsedimented material was removed by slowly pipetting out 0.5 mL of medium by using a 1-mL pipette tip placed above the center of the coverslip, carefully leaving enough medium to cover cells attached onto the coverslip and removing all medium at the wall of the plate. Cells were left overnight in 0.5 mL complete B27 Plus Neurob-A containing 10 ng/mL FGF1, 10 ng/mL FGF2, 0.5 mg/mL vitamin D3, 20 nM MTII, Penicillin, 100 IU and Streptomycin 100 μg/mL (complete B27 Plus Neuro P/S). Medium with the remaining unsedimented material was removed by pipetting, as described above. Then, 1 mL of complete B27 Plus Neurob-A P/S was added to the plate. Cells were kept in culture for 10–14 days by replacing 0.5 mL of the medium with 0.5 mL fresh medium twice per week. On Day 8–12 after plating, cells were incubated for 48 h with a final concentration of 250 µM palmitate, with or without 100 nM liraglutide or 2 mM metformin in complete B27 Plus Neuro P/S.

### 4.7. Monitoring Mitochondrial Size and MMP in Hypothalamic Neurons

To monitor mitochondrial size and MMP under different conditions, complete B27 Plus Neuro P/S containing no additions, 250 µM palmitate, with or without 100 nM liraglutide or 2 mM metformin, was removed to leave behind 200 µL to cover the bottom of the plate. MitoTracker Red was added to a final concentration of 200 nM and the plates were transferred to the tissue-culture cabinet for 15 min. Cells were washed by adding to the side of the plate 1 mL of Neurob-A P/S medium, which was removed with the exception of 200 µL to cover the bottom of the plate. Fresh complete B27 Plus Neurob-A P/S (1.5 mL) replicating the individual experimental conditions was added to each plate. 

Mitochondrial size. Cells bearing processes were imaged at room temperature using the 60× objective of the confocal Olympus FV1000 microscope with the appropriate laser/bandpass filter selected by choosing “MitoTracker Red” from the dye list. Images were opened in NIH software ImageJ. All images were thresholded by using the commands *Image > Adjust > Threshold*. Thresholds were set with identical parameters for all images. Parameters of measurements were set by using the command “*Analyze > Measure*” and selecting “*Area*” from the list. The areas of mitochondria were measured in ROIs that include at least two neuronal processes per neuron. Analysis was done using, on the “*Analyze Particle*” menu, the commands “*Display Results*”, “*Summarize*”, “*Add to Manager*”. In the “*Summary*”, the “*Average Areas*” of mitochondria were derived from approximately 50–100 particles per neuronal process. 

*MMP.* ROIs were created to include the cell bodies of the neurons. MitoTracker fluorescence intensity per cell was measured by selecting the command “*Set Measurement > Integrated Density*” and then “*Analyze > Measure*” to measure the “*Raw Integrated Density*” (sum of pixel values) of the selected ROIs.

### 4.8. Statistical Analysis

All statistical analyses were performed using GraphPad Prism 6 software. Statistical significance was calculated by two-way ANOVA with Turkey multiple comparison test on multiple groups. A value of *p* < 0.05 was considered statistically significant. Data were expressed as mean ± S.D. Results for Neuro2A cells were derived using at least 3–4 Petri plates in 3–4 independent assays (n > 25–50 cells) for each condition. Results for hypothalamic neurons were derived using at least 2–3 Petri plates in 2–3 independent assays (n > 9–15 differentiated neurons) for each condition.

## Data Availability

Not applicable.

## References

[1] Thaler J.P., Yi C.X., Schur E.A., Guyenet S.J., Hwang B.H., Dietrich M.O., Zhao X., Sarruf D.A., Izgur V., Maravilla K.R. (2012). Obesity is associated with hypothalamic injury in rodents and humans. J. Clin. Investig..

[2] De Souza C.T., Araujo E.P., Bordin S., Ashimine R., Zollner R.L., Boschero A.C., Saad M.J.A., Velloso L.A. (2005). Consumption of a fat-rich diet activates a proinflammatory response and induces insulin resistance in the hypothalamus. Endocrinology.

[3] Valdearcos M., Robblee M.M., Benjamin D.I., Nomura D.K., Xu A.W., Koliwad S.K. (2014). Microglia dictate the impact of saturated fat consumption on hypothalamic inflammation and neuronal function. Cell Rep..

[4] Leon S., Nadjar A., Quarta C. (2021). Microglia-Neuron Crosstalk in Obesity: Melodious Interaction or Kiss of Death?. Int. J. Mol. Sci..

[5] Cakir I., Cyr N.E., Perello M., Litvinov B.P., Romero A., Stuart R.C., Nillni E.A. (2013). Obesity induces hypothalamic endoplasmic reticulum stress and impairs proopiomelanocortin (POMC) post-translational processing. J. Biol. Chem..

[6] Nillni E.A. (2007). Regulation of prohormone convertases in hypothalamic neurons: Implications for prothyrotropin-releasing hormone and proopiomelanocortin. Endocrinology.

[7] Nyamugenda E., Griffin H., Russell S., Cooney K.A., Kowalczyk N.S., Islam I., Phelan K.D., Baldini G. (2020). Selective Survival of Sim1/MC4R Neurons in Diet-Induced Obesity. iScience.

[8] Nyamugenda E., Trentzsch M., Russell S., Miles T., Boysen G., Phelan K.D., Baldini G. (2019). Injury to hypothalamic Sim1 neurons is a common feature of obesity by exposure to high-fat diet in male and female mice. J. Neurochem..

[9] Melo H.M., Seixas da Silva G.D.S., Sant’Ana M.R., Teixeira C.V.L., Clarke J.R., Miya Coreixas V.S., de Melo B.C., Fortuna J.T.S., Forny-Germano L., Ledo J.H. (2020). Palmitate Is Increased in the Cerebrospinal Fluid of Humans with Obesity and Induces Memory Impairment in Mice via Pro-inflammatory TNF-alpha. Cell Rep..

[10] Guttenplan K.A., Weigel M.K., Prakash P., Wijewardhane P.R., Hasel P., Rufen-Blanchette U., Munch A.E., Blum J.A., Fine J., Neal M.C. (2021). Neurotoxic reactive astrocytes induce cell death via saturated lipids. Nature.

[11] Belegri E., Rijnsburger M., Eggels L., Unmehopa U., Scheper W., Boelen A., la Fleur S.E. (2017). Effects of Fat and Sugar, Either Consumed or Infused toward the Brain, on Hypothalamic ER Stress Markers. Front. Neurosci..

[12] Cragle F.K., Baldini G. (2014). Mild lipid stress induces profound loss of MC4R protein abundance and function. Mol. Endocrinol..

[13] Mayer C.M., Belsham D.D. (2010). Palmitate attenuates insulin signaling and induces endoplasmic reticulum stress and apoptosis in hypothalamic neurons: Rescue of resistance and apoptosis through adenosine 5′ monophosphate-activated protein kinase activation. Endocrinology.

[14] Ramirez S., Claret M. (2015). Hypothalamic ER stress: A bridge between leptin resistance and obesity. FEBS Lett..

[15] Han J., Kaufman R.J. (2016). The role of ER stress in lipid metabolism and lipotoxicity. J. Lipid Res..

[16] Jantti M.H., Jackson S.N., Kuhn J., Parkkinen I., Sree S., Hinkle J.J., Jokitalo E., Deterding L.J., Harvey B.K. (2022). Palmitate and thapsigargin have contrasting effects on ER membrane lipid composition and ER proteostasis in neuronal cells. Biochim. Biophys. Acta Mol. Cell Biol. Lipids.

[17] Trentzsch M., Nyamugenda E., Miles T.K., Griffin H., Russell S., Koss B., Cooney K.A., Phelan K.D., Tackett A.J., Iyer S. (2020). Delivery of phosphatidylethanolamine blunts stress in hepatoma cells exposed to elevated palmitate by targeting the endoplasmic reticulum. Cell Death Discov..

[18] Hentges S.T., Nishiyama M., Overstreet L.S., Stenzel-Poore M., Williams J.T., Low M.J. (2004). GABA release from proopiomelanocortin neurons. J. Neurosci..

[19] Schneeberger M., Dietrich M.O., Sebastian D., Imbernon M., Castano C., Garcia A., Esteban Y., Gonzalez-Franquesa A., Rodriguez I.C., Bortolozzi A. (2013). Mitofusin 2 in POMC neurons connects ER stress with leptin resistance and energy imbalance. Cell.

[20] Zorzano A., Claret M. (2015). Implications of mitochondrial dynamics on neurodegeneration and on hypothalamic dysfunction. Front. Aging Neurosci..

[21] Rumora A.E., LoGrasso G., Hayes J.M., Mendelson F.E., Tabbey M.A., Haidar J.A., Lentz S.I., Feldman E.L. (2019). The Divergent Roles of Dietary Saturated and Monounsaturated Fatty Acids on Nerve Function in Murine Models of Obesity. J. Neurosci..

[22] Jo D., Yoon G., Song J. (2021). Role of Exendin-4 in Brain Insulin Resistance, Mitochondrial Function, and Neurite Outgrowth in Neurons under Palmitic Acid-Induced Oxidative Stress. Antioxidants.

[23] American Diabetes Association (2019). 8. Obesity Management for the Treatment of Type 2 Diabetes: Standards of Medical Care in Diabetes—2019. Diabetes Care.

[24] le Roux C.W., Astrup A., Fujioka K., Greenway F., Lau D.C.W., Van Gaal L., Ortiz R.V., Wilding J.P.H., Skjoth T.V., Manning L.S. (2017). 3 years of liraglutide versus placebo for type 2 diabetes risk reduction and weight management in individuals with prediabetes: A randomised, double-blind trial. Lancet.

[25] Ly L.D., Xu S., Choi S.K., Ha C.M., Thoudam T., Cha S.K., Wiederkehr A., Wollheim C.B., Lee I.K., Park K.S. (2017). Oxidative stress and calcium dysregulation by palmitate in type 2 diabetes. Exp. Mol. Med..

[26] Bommiasamy H., Back S.H., Fagone P., Lee K., Meshinchi S., Vink E., Sriburi R., Frank M., Jackowski S., Kaufman R.J. (2009). ATF6alpha induces XBP1-independent expansion of the endoplasmic reticulum. J. Cell Sci..

[27] Liu J., Wei L., Wang Z., Song S., Lin Z., Zhu J., Ren X., Kong L. (2020). Protective effect of Liraglutide on diabetic retinal neurodegeneration via inhibiting oxidative stress and endoplasmic reticulum stress. Neurochem. Int..

[28] Xu S., Nam S.M., Kim J.H., Das R., Choi S.K., Nguyen T.T., Quan X., Choi S.J., Chung C.H., Lee E.Y. (2015). Palmitate induces ER calcium depletion and apoptosis in mouse podocytes subsequent to mitochondrial oxidative stress. Cell Death Dis..

[29] Baldini G., Phelan K.D. (2019). The melanocortin pathway and control of appetite-progress and therapeutic implications. J. Endocrinol..

[30] Granell S., Mohammad S., Ramanagoudr-Bhojappa R., Baldini G. (2010). Obesity-linked variants of melanocortin-4 receptor are misfolded in the endoplasmic reticulum and can be rescued to the cell surface by a chemical chaperone. Mol. Endocrinol..

[31] D’Andrea M.R., Howanski R.J., Saller C.F. (2017). MAP2 IHC detection: A marker of antigenicity in CNS tissues. Biotech. Histochem..

[32] McCall M.A., Gregg R.G., Behringer R.R., Brenner M., Delaney C.L., Galbreath E.J., Zhang C.L., Pearce R.A., Chiu S.Y., Messing A. (1996). Targeted deletion in astrocyte intermediate filament (Gfap) alters neuronal physiology. Proc. Natl. Acad. Sci. USA.

[33] Ito D., Imai Y., Ohsawa K., Nakajima K., Fukuuchi Y., Kohsaka S. (1998). Microglia-specific localisation of a novel calcium binding protein, Iba1. Mol. Brain Res..

[34] Parato J., Bartolini F. (2021). The microtubule cytoskeleton at the synapse. Neurosci. Lett..

[35] Jeong J.H., Lee D.K., Jo Y.H. (2017). Cholinergic neurons in the dorsomedial hypothalamus regulate food intake. Mol. Metab..

[36] Holder J.L., Butte N.F., Zinn A.R. (2000). Profound obesity associated with a balanced translocation that disrupts the SIM1 gene. Hum. Mol. Genet..

[37] Cakir I., Diaz-Martinez M., Lining Pan P., Welch E.B., Patel S., Ghamari-Langroudi M. (2019). Leptin Receptor Signaling in Sim1-Expressing Neurons Regulates Body Temperature and Adaptive Thermogenesis. Endocrinology.

[38] Rossi J., Balthasar N., Olson D., Scott M., Berglund E., Lee C.E., Choi M.J., Lauzon D., Lowell B.B., Elmquist J.K. (2011). Melanocortin-4 receptors expressed by cholinergic neurons regulate energy balance and glucose homeostasis. Cell Metab..

[39] Muntean B.S., Zucca S., MacMullen C.M., Dao M.T., Johnston C., Iwamoto H., Blakely R.D., Davis R.L., Martemyanov K.A. (2018). Interrogating the Spatiotemporal Landscape of Neuromodulatory GPCR Signaling by Real-Time Imaging of cAMP in Intact Neurons and Circuits. Cell Rep..

[40] Choi S.J., Kim F., Schwartz M.W., Wisse B.E. (2010). Cultured hypothalamic neurons are resistant to inflammation and insulin resistance induced by saturated fatty acids. Am. J. Physiol. Endocrinol. Metab..

[41] Trychta K.A., Back S., Henderson M.J., Harvey B.K. (2018). KDEL Receptors Are Differentially Regulated to Maintain the ER Proteome under Calcium Deficiency. Cell Rep..

[42] Wires E.S., Trychta K.A., Back S., Sulima A., Rice K.C., Harvey B.K. (2017). High fat diet disrupts endoplasmic reticulum calcium homeostasis in the rat liver. J. Hepatol..

[43] Park S.W., Zhou Y., Lee J., Ozcan U. (2010). Sarco(endo)plasmic reticulum Ca^2+^-ATPase 2b is a major regulator of endoplasmic reticulum stress and glucose homeostasis in obesity. Proc. Natl. Acad. Sci. USA.

[44] Hojmann Larsen A., Frandsen A., Treiman M. (2001). Upregulation of the SERCA-type Ca^2+^ pump activity in response to endoplasmic reticulum stress in PC12 cells. BMC Biochem..

[45] Hroudova J., Singh N., Fisar Z. (2014). Mitochondrial dysfunctions in neurodegenerative diseases: Relevance to Alzheimer’s disease. Biomed. Res. Int..

[46] Molina A.J., Wikstrom J.D., Stiles L., Las G., Mohamed H., Elorza A., Walzer G., Twig G., Katz S., Corkey B.E. (2009). Mitochondrial networking protects beta-cells from nutrient-induced apoptosis. Diabetes.

[47] Wang Q., Zhang M., Torres G., Wu S., Ouyang C., Xie Z., Zou M.H. (2017). Metformin Suppresses Diabetes-Accelerated Atherosclerosis via the Inhibition of Drp1-Mediated Mitochondrial Fission. Diabetes.

[48] Buckman J.F., Hernandez H., Kress G.J., Votyakova T.V., Pal S., Reynolds I.J. (2001). MitoTracker labeling in primary neuronal and astrocytic cultures: Influence of mitochondrial membrane potential and oxidants. J. Neurosci. Methods.

[49] Simon-Szabo L., Kokas M., Mandl J., Keri G., Csala M. (2014). Metformin attenuates palmitate-induced endoplasmic reticulum stress, serine phosphorylation of IRS-1 and apoptosis in rat insulinoma cells. PLoS ONE.

[50] Kapadia P., Bikkina P., Landicho M.A., Parekh S., Haas M.J., Mooradian A.D. (2021). Effect of anti-hyperglycemic drugs on endoplasmic reticulum (ER) stress in human coronary artery endothelial cells. Eur. J. Pharmacol..

[51] Diaz-Morales N., Iannantuoni F., Escribano-Lopez I., Banuls C., Rovira-Llopis S., Sola E., Rocha M., Hernandez-Mijares A., Victor V.M. (2018). Does Metformin Modulate Endoplasmic Reticulum Stress and Autophagy in Type 2 Diabetic Peripheral Blood Mononuclear Cells?. Antioxid. Redox Signal..

[52] Oslowski C.M., Urano F. (2011). Measuring ER stress and the unfolded protein response using mammalian tissue culture system. Methods Enzymol..

[53] Granell S., Baldini G., Mohammad S., Nicolin V., Narducci P., Storrie B., Baldini G. (2008). Sequestration of Mutated α1-Antitrypsin into Inclusion Bodies Is a Cell-protective Mechanism to Maintain Endoplasmic Reticulum Function. Mol. Biol. Cell.

[54] Li H., Sun S. (2021). Protein Aggregation in the ER: Calm behind the Storm. Cells.

[55] Loi M., Raimondi A., Morone D., Molinari M. (2019). ESCRT-III-driven piecemeal micro-ER-phagy remodels the ER during recovery from ER stress. Nat. Commun..

[56] Balthasar N., Dalgaard L.T., Lee C.E., Yu J., Funahashi H., Williams T., Ferreira M., Tang V., McGovern R.A., Kenny C.D. (2005). Divergence of melanocortin pathways in the control of food intake and energy expenditure. Cell.

[57] Gabery S., Salinas C.G., Paulsen S.J., Ahnfelt-Ronne J., Alanentalo T., Baquero A.F., Buckley S.T., Farkas E., Fekete C., Frederiksen K.S. (2020). Semaglutide lowers body weight in rodents via distributed neural pathways. JCI Insight.

[58] Sisley S., Gutierrez-Aguilar R., Scott M., D’Alessio D.A., Sandoval D.A., Seeley R.J. (2014). Neuronal GLP1R mediates liraglutide’s anorectic but not glucose-lowering effect. J. Clin. Investig..

[59] Secher A., Jelsing J., Baquero A.F., Hecksher-Sorensen J., Cowley M.A., Dalboge L.S., Hansen G., Grove K.L., Pyke C., Raun K. (2014). The arcuate nucleus mediates GLP-1 receptor agonist liraglutide-dependent weight loss. J. Clin. Investig..

[60] Guerrero-Hernandez A., Leon-Aparicio D., Chavez-Reyes J., Olivares-Reyes J.A., DeJesus S. (2014). Endoplasmic reticulum stress in insulin resistance and diabetes. Cell Calcium.

[61] Prell T., Lautenschlager J., Grosskreutz J. (2013). Calcium-dependent protein folding in amyotrophic lateral sclerosis. Cell Calcium.

[62] Fu S., Yang L., Li P., Hofmann O., Dicker L., Hide W., Lin X., Watkins S.M., Ivanov A.R., Hotamisligil G.S. (2011). Aberrant lipid metabolism disrupts calcium homeostasis causing liver endoplasmic reticulum stress in obesity. Nature.

[63] Sims S.G., Cisney R.N., Lipscomb M.M., Meares G.P. (2022). The role of endoplasmic reticulum stress in astrocytes. GLIA.

[64] Valdearcos M., Douglass J.D., Robblee M.M., Dorfman M.D., Stifler D.R., Bennett M.L., Gerritse I., Fasnacht R., Barres B.A., Thaler J.P. (2017). Microglial Inflammatory Signaling Orchestrates the Hypothalamic Immune Response to Dietary Excess and Mediates Obesity Susceptibility. Cell Metab..

[65] Dorfman M.D., Krull J.E., Douglass J.D., Fasnacht R., Lara-Lince F., Meek T.H., Shi X., Damian V., Nguyen H.T., Matsen M.E. (2017). Sex differences in microglial CX3CR1 signalling determine obesity susceptibility in mice. Nat. Commun..

[66] Kuo Y.T., So P.W., Parkinson J.R., Yu W.S., Hankir M., Herlihy A.H., Goldstone A.P., Frost G.S., Wasserfall C., Bell J.D. (2010). The combined effects on neuronal activation and blood-brain barrier permeability of time and n-3 polyunsaturated fatty acids in mice, as measured in vivo using MEMRI. Neuroimage.

[67] Song J.E., Alves T.C., Stutz B., Sestan-Pesa M., Kilian N., Jin S., Diano S., Kibbey R.G., Horvath T.L. (2021). Mitochondrial Fission Governed by Drp1 Regulates Exogenous Fatty Acid Usage and Storage in Hela Cells. Metabolites.

[68] Jayashankar V., Selwan E., Hancock S.E., Verlande A., Goodson M.O., Eckenstein K.H., Milinkeviciute G., Hoover B.M., Chen B., Fleischman A.G. (2021). Drug-like sphingolipid SH-BC-893 opposes ceramide-induced mitochondrial fission and corrects diet-induced obesity. EMBO Mol. Med..

[69] Toda C., Kim J.D., Impellizzeri D., Cuzzocrea S., Liu Z.W., Diano S. (2016). UCP2 Regulates Mitochondrial Fission and Ventromedial Nucleus Control of Glucose Responsiveness. Cell.

[70] Liesa M., Shirihai O.S. (2013). Mitochondrial dynamics in the regulation of nutrient utilization and energy expenditure. Cell Metab..

[71] Timper K., Paeger L., Sanchez-Lasheras C., Varela L., Jais A., Nolte H., Vogt M.C., Hausen A.C., Heilinger C., Evers N. (2018). Mild Impairment of Mitochondrial OXPHOS Promotes Fatty Acid Utilization in POMC Neurons and Improves Glucose Homeostasis in Obesity. Cell Rep..

[72] Wicinski M., Socha M., Malinowski B., Wodkiewicz E., Walczak M., Gorski K., Slupski M., Pawlak-Osinska K. (2019). Liraglutide and its Neuroprotective Properties-Focus on Possible Biochemical Mechanisms in Alzheimer’s Disease and Cerebral Ischemic Events. Int. J. Mol. Sci..

[73] Dai Y., Dai D., Wang X., Ding Z., Li C., Mehta J.L. (2014). GLP-1 agonists inhibit ox-LDL uptake in macrophages by activating protein kinase A. J. Cardiovasc. Pharmacol..

[74] Le Foll C., Dunn-Meynell A., Musatov S., Magnan C., Levin B.E. (2013). FAT/CD36: A major regulator of neuronal fatty acid sensing and energy homeostasis in rats and mice. Diabetes.

[75] Urso C.J., Zhou H. (2021). Role of CD36 in Palmitic Acid Lipotoxicity in Neuro-2a Neuroblastoma Cells. Biomolecules..

[76] Zeeshan H.M., Lee G.H., Kim H.R., Chae H.J. (2016). Endoplasmic Reticulum Stress and Associated ROS. Int. J. Mol. Sci..

[77] Ochoa C.D., Wu R.F., Terada L.S. (2018). ROS signaling and ER stress in cardiovascular disease. Mol. Asp. Med..

[78] McNay D.E.G., Briancon N., Kokoeva M.V., Maratos-Flier E., Flier J.S. (2012). Remodeling of the arcuate nucleus energy-balance circuit is inhibited in obese mice. J. Clin. Investig..

[79] Moraes J.C., Coope A., Morari J., Cintra D.E., Roman E.A., Pauli J.R., Romanatto T., Carvalheira J.B., Oliveira A.L., Saad M.J. (2009). High-fat diet induces apoptosis of hypothalamic neurons. PLoS ONE.

[80] Mohammad S., Baldini G., Granell S., Narducci P., Martelli A.M., Baldini G. (2007). Constitutive traffic of melanocortin-4 receptor in Neuro2A cells and immortalized hypothalamic neurons. J. Biol. Chem..

